# Supplementary material for: CHID1 Is a Novel Prognostic Marker of Non-Small Cell Lung Cancer
Source: Int J Mol Sci. 2021 Jan 5;22(1):450. doi: 10.3390/ijms22010450 (PMC7795388; doi:10.3390/ijms22010450)
Supplement: Supplementary file 1 [file ijms-22-00450-s001.pdf]

Supplementary table 1.

	CD3			p	CD8				p	FOXP3				p
	low	medium	high		0	low	medium	high		0	low	medium	high	
Histology				0,705					0,768					0,0005*
AC	11	16	24		1	26	13	11		17	25	6	3	
SCC	13	14	22		1	23	14	11		7	17	16	9	
Location				0,145					0,632					0,115
central	8	11	23		2	18	11	11		13	18	7	4	
peripheral	16	19	23		0	31	16	11		11	24	15	8	
Stage				0,038*					0,011*					0,430
I-II	9	12	28		2	18	11	18		15	18	9	7	
III-IV	15	18	18		0	31	16	4		9	24	13	5	
Grade (G)				0,700					0,7125					0,946
G1-G2 (high)	13	19	28		0	30	17	13		14	25	15	6	
G3-G4 (low)	11	11	18		2	19	10	9		10	17	7	6	
Nodal status (N)				0,265					0,059					0,131
N0	7	9	19		1	13	9	12		13	12	5	5	
N+	17	21	27		1	36	18	10		11	30	17	7	
Tumor size (T)				0,012*					0,005*					0,348
1-2	10	13	32		2	20	14	19		15	23	11	6	
3-4	14	17	14		0	29	13	3		9	19	11	6	

Supplementary table 2

	CD68			p	CD163			p	CD206				p
	Low	medium	high		low	medium	high		0	low	medium	high	
Histology				0,116				0,247					0,954
AC	4	25	22		9	23	19		0	21	15	15	
SCC	5	13	31		10	12	27		1	22	8	18	
Location				0,244				0,960					0,755
central	5	19	34		12	19	27		1	27	9	21	
peripheral	4	19	19		7	16	19		0	16	14	12	
Stage				0,509				>0,999					0,338
I-II	6	18	25		10	16	23		1	17	14	17	
III-IV	3	20	28		9	19	23		0	26	9	16	
Grade (G)				0,719				>0,999					0,084
G1-G2 (high)	4	26	30		12	20	28		1	20	17	22	
G3-G4 (low)	5	12	23		7	15	18		0	23	6	11	
Nodal status (N)				0,214				0,429					0,406
N0	5	14	16		9	11	15		1	11	11	12	
N+	4	24	37		10	24	31		0	32	12	21	
Tumor size (T)				0,989				0,512					0,376
1-2	5	21	29		8	21	26		0	20	18	17	
3-4	4	17	24		11	14	20		1	23	5	16	

	CD204			p	CHID1			p
	low	medium	high		low	medium	high	
Histology				0,086				0,156
AC	15	21	15		10	18	23	
SCC	12	12	25		6	14	29	
Location				0,185				0,524
central	13	19	26		7	11	24	
peripheral	14	14	14		9	21	28	
Stage				0,609				0,259
I-II	15	15	19		6	15	28	
III-IV	12	18	21		10	17	24	
Grade (G)				0,037*				0,992
G1-G2 (high)	13	18	29		10	19	31	
G3-G4 (low)	14	15	11		6	13	21	
Nodal status (N)				0,431				0,858
N0	13	8	14		6	10	19	
N+	14	25	26		10	22	33	
Tumor size (T)				0,569				0,133
1-2	15	20	20		5	19	31	
3-4	12	13	20		11	13	21	
